# Behavioral Defects in Chaperone-Deficient Alzheimer's Disease Model Mice

**DOI:** 10.1371/journal.pone.0016550

**Published:** 2011-02-17

**Authors:** Juhi Ojha, Rajalakshmi V. Karmegam, J. Gunasingh Masilamoni, Alvin V. Terry, Anil G. Cashikar

**Affiliations:** 1 Center for Molecular Chaperones and Radiobiology, Medical College of Georgia, Augusta, Georgia, United States of America; 2 Department of Pharmacology and Toxicology, Medical College of Georgia, Augusta, Georgia, United States of America; St. Georges University of London, United Kingdom

## Abstract

Molecular chaperones protect cells from the deleterious effects of protein misfolding and aggregation. Neurotoxicity of amyloid-beta (Aβ) aggregates and their deposition in senile plaques are hallmarks of Alzheimer's disease (AD). We observed that the overall content of αB-crystallin, a small heat shock protein molecular chaperone, decreased in AD model mice in an age-dependent manner. We hypothesized that αB-crystallin protects cells against Aβ toxicity. To test this, we crossed αB-crystallin/HspB2 deficient (*CRYAB^-/-^HSPB2^-/-^*) mice with AD model transgenic mice expressing mutant human amyloid precursor protein. Transgenic and non-transgenic mice in chaperone-sufficient or deficient backgrounds were examined for representative behavioral paradigms for locomotion and memory network functions: (i) spatial orientation and locomotion was monitored by open field test; (ii) sequential organization and associative learning was monitored by fear conditioning; and (iii) evoked behavioral response was tested by hot plate method. Interestingly, αB-crystallin/HspB2 deficient transgenic mice were severely impaired in locomotion compared to each genetic model separately. Our results highlight a synergistic effect of combining chaperone deficiency in a transgenic mouse model for AD underscoring an important role for chaperones in protein misfolding diseases.

## Introduction

Accumulation of misfolded and aggregated proteins is a pathological hallmark of several neurodegenerative diseases. In Alzheimer's disease (AD), aggregated amyloid-beta peptide (Aβ) is a primary component of the senile plaques and is thought to be central to the associated neurotoxicity. Because molecular chaperones have evolved to protect cells against protein misfolding and aggregation, their importance in protein aggregation diseases must be understood. Members of the small heat shock proteins (sHsps) family, whose expression is regulated by the heat shock transcription factor 1 (HSF1), protect cells from a variety of environmental conditions such as heat and oxidative stress by antagonizing protein aggregation. *In vivo*, sHsps play an important role in enhancing stress resistance, regulating actin and intermediate filament dynamics, and inhibiting apoptosis [1], [2]. sHsps share a conserved α-crystallin domain of 80–100 amino acids at their C-terminus whereas their N-terminal regions are highly variable in sequence and length [3], [4]. The sHsps form dynamic oligomeric structures ranging from 9–50 subunits associating as either homo- or hetero-multimeric complexes [5], [6]. It has been proposed that the ATP-independent sHsps aid in refolding of denatured proteins by holding them in a reactivation-competent state and target them to subsequent refolding or degradation with the help of ATP-dependent chaperones like Hsp70 [2], [7].

The human and mouse genomes code for 10 genes for sHsps differing between 45 and 85% in sequence [8], [9]. Of these Hsp27 (Hsp25 in mouse), αB-crystallin, HspB6 and HspB8 are ubiquitously expressed [8]. Point mutations in human sHsps lead to several aggregation diseases [10] – for example, mutations in αA-crystallin leads to cataract [11], mutations in αB-crystallin leads to desmin-related myopathy [12], [13], [14], missense mutations in HSP27 is associated with Charcot-Marie-Tooth disease [15]. Over-expression of Hsp27 is highly protective against toxicity induced by αSyn [16] or polyglutamine [17] in cell culture models. Hsp27 and αB-crystallin have been found in proteinaceous inclusions of Alzheimer's and Parkinson's disease [18], [19], [20], [21], [22], [23], [24]. The sHsps are associated with senescence and longevity in worms [25], [26] and flies [27] suggesting their importance in aging-related diseases. Induction of αB-crystallin has been noted in Alexander's disease [28], Creutzfeldt-Jacob disease [22] and Alzheimer's disease [29]. Interestingly, the amyloid precursor protein (APP) central to AD was found to interact with αB-crystallin in worm [30] and mammalian cell models [31]. These observations underscore the importance of αB-crystallin (sHsps in general) in diseases of aging and protein aggregation, but their importance in mouse models has not been examined. To this end, we examined behavioral deficits when AD model mice were crossed mice lacking αB-crystallin/HspB2.

Mice knocked-out for αB-crystallin and HspB2 (genes are *CRYAB* and *HSPB2*, respectively) [32] are viable suggesting that it is either non-essential or redundant during development. Due to the close proximity (<1 kb) of the *HSPB2* gene to *CRYAB* and the sharing of promoter elements [33], the αB-crystallin knockout mice are also deficient for a second sHsp, HspB2. The knockout mice showed no significant difference in development and growth in comparison to wild-type mice until 40 weeks of age. Older knockout mice consistently lost weight and body fat and subsequently developed severe spine curvature (kyphosis) and skeletal muscle degeneration [32].

The importance of sHsps in mice experiencing an increased proteotoxic stress was previously not studied. In this report, we inter-crossed between mice lacking αB-crystallin/HspB2 [32] and Tg2576 transgenic mice [34], which over-produces the highly aggregation-prone amyloid-beta. We observed that chaperone-deficient transgenic mice developed severe locomotion defects compared to mice with either chaperone deficiency alone or transgene expression alone. Our results show that exacerbation of protein aggregation and loss of chaperones produced a new synthetic phenotype of motor defects in mice.

## Materials and Methods

### Mice and Animal Care

One male B6;SJL-Tg(APPSWE2576Kha) transgenic mouse (Tg2576) [34] was purchased from Taconic Farms. The Tg2576 mice over-expresses the 695 amino acid isoform of amyloid precursor protein (APP) harboring Swedish familial mutations (K670N, M67IL) under the PrP promoter. These mice show impairment in learning and behavior by 9 months and develop extracellular amyloid plaques by 12 months. This mouse was out-crossed to 129SvEv wild-type mice for 4 generations. The resulting litters contained non-transgenic or transgenic mice. One breeding pair of *CRYAB^-/-^/HSPB2^-/-^* mice in 129SvEv background [32] were gifted kindly by Dr. Eric Wawrousek, National Eye Institute, NIH, Bethesda, MD. Mice were maintained in a facility and program accredited by the Association for Assessment and Accreditation of Laboratory Animal Care International (AAALAC) with the approval number A3307-01. The animal use protocols were authorized by the Institutional Animal Care and Use Committee (IACUC) of the Medical College of Georgia.

All behavioral experiments were carried out at the Small Animal Behavior Core facility of the Medical College of Georgia in rooms equipped with white noise generators (San Diego Instruments, San Diego, CA) set to provide a constant background level of 70 dB and ambient lighting of approximately 25–30 Lux (lumen/m^2^). Test subjects were handled daily for several minutes (each) for at least one week prior to experimentation. Animals were transferred (in their home cages) to the behavioral testing rooms each morning approximately 30 min before the beginning of experiments. Measures were taken to minimize pain or discomfort in accordance with the National Institute of Health Guide for the Care and Use of Laboratory Animals (NIH Publications No. 80-23) revised 1996. Significant efforts were also made to minimize the total number of animals used while maintaining statistically valid group numbers. All procedures employed during this study were reviewed and approved by the Medical College of Georgia IACUC and are consistent with AAALAC guidelines.

### Generation of the required genotypes

To generate αB-crystallin/HspB2 deficient mice expressing the transgene, transgenic mice in 129SvEv background were crossed with the *CRYAB^-/-^* mice. The resulting litters contained *CRYAB^+/-^/HSPB2^+/-^,* mice with equal numbers of non-transgenics and transgenics. The *CRYAB^+/-^/HSPB2^+/-^* transgenic mice were crossed with *CRYAB^+/-^/HSPB2^+/-^* littermates to generate (i) *CRYAB^+/+^/HSPB2^+/+^, Tg^0/0^* (hereafter referred to as WT); (ii) *CRYAB^+/+^/HSPB2^+/+^, Tg^+/0^* (hereafter referred to as WTTg); (iii) *CRYAB^-/-^/HSPB2^-/-^, Tg^0/0^* (hereafter referred to as KO) and (iv) *CRYAB^-/-^/HSPB2^-/-^, Tg^+/0^* (hereafter referred to as KOTg) in addition to parental genotypes *CRYAB^+/-^/HSPB2^+/-^, Tg^0/0^* and *CRYAB^+/-^/HSPB2^+/-^, Tg^+/0^*, which were not used further. Genotypes of the mice were confirmed by PCR of tail genomic DNA using appropriate primers as described in Brady et al [32] or in Hsiao et al [34]. The use of littermates for all genotypes minimizes the effect of out-crossing the mice. The genetic variations between the two parental mouse strains are expected to affect all four experimental genotypes identically.

### Immunoblotting

To prepare brain lysates mice were euthanized using carbon dioxide followed by perfusion with PBS. Brains were dissected out from appropriate mice, frozen on liquid nitrogen and stored at -80°C until use. Lysates were prepared by homogenization in lysis buffer containing 50 mM Tris.HCl pH 7.5, 5 mM EDTA, 1% Triton-X100 supplemented with HALT protease inhibitor cocktail (Pierce). Lysate were cleared by centrifugation and the protein concentration was estimated using the BCA kit (Pierce). Samples were boiled in loading dye, separated by SDS-PAGE and transferred to PVDF membrane. The membrane was blocked using 10% milk in PBS with 0.1% tween-20. Primary antibodies were anti-Hsp27 (Sigma), Anti-αBcrystallin (Stressgen), anti-Hsp70 (Stressgen) and anti-Aβ (Sigma). Appropriate secondary antibodies (rabbit anti-mouse or goat anti-rabbit) conjugated with horseradish peroxidase were used followed by development of the immunoblot by chemiluminescence (Danville).

### Open Field Activity

Mouse open field activity monitors (27.9 cm ×27.9 cm, Med Associates St Albans, VT) were used for these experiments. The following parameters were recorded for the 25 min test session: horizontal activity (horizontal photobeam breaks or counts), number of stereotypical movements, and vertical activity (vertical photobeam breaks). Thus, spontaneous locomotor activity, olfactory activity (rearing and sniffing movements) and stereotypical movements were assessed.

### Hot Plate test (Supraspinal Nociception)

Individual mice were placed in the glass enclosed section of a Hot-Plate Analgesia Meter, Accuscan Instruments, Inc., Columbus OH. The temperature of the heating surface was elevated by 3°C per minute from a beginning temperature of 42°C to a maximum temperature of 49°C. The time elapsed (latency) before the subject lifted and/or licked a hind paw or jumped was recorded as a measure of nociception. Each mouse was given 3 trials separated by a 30-minute (minimum) inter-trial interval.

### Fear Conditioning

#### Shock Threshold

A sequence of single foot shocks were delivered to wild type mouse subjects placed on the same electrified grid used for fear conditioning (see below) in order to assess the shock (i.e., sensory perception) threshold. Initially, a 0.1-mV shock was delivered for 1 sec, and the animals' behavior was evaluated for flinching, jumping, and vocalization. At 30-s intervals the shock intensity was increased by 0.1 mA up to 0.7 mA and then returned to 0 mA in 0.1-mA increments at 30-sec intervals. Threshold to vocalization, flinching, and then jumping were quantified for each animal by averaging the shock intensity at which each animal manifested a behavioral response to the foot shock. This average was determined to be 0.5 mA and used in subsequent experiments.

#### Training procedure

Separate groups of mice were subsequently evaluated for contextual and cued fear conditioning using the mA shock levels determined in the experiments described above. A two-pairing method of auditory cue and mild foot shock were conducted during a 7 min test session. Animals were initially allowed to freely explore the apparatus (MED-VFC-NIR-M, Med Associates, St Albans, VT) for 3 min, after which a 30-sec acoustic-conditioned stimulus (CS; white noise, 80 dB) was delivered. At the end of the CS, a 2-sec shock unconditioned stimulus (US) was applied to the grid floor. The CS-US pairing was delivered again at the 5-min mark.

#### Context-dependent Freezing

To evaluate contextual fear learning, test subjects were returned to the freeze monitor (i.e., same context) 24 hr after the training procedure described above, and freezing behavior was scored for 5 min.

#### Cue-dependent Freezing

To evaluate cued fear learning, the animals were placed in a different context (novel odor, lighting, cage floor, and visual cues) 24 hours following contextual testing. Baseline behavior was scored for 2 minutes, and then the CS was presented for a period of 2 minutes, followed by another 2 minutes period in which the auditory stimulus was absent.

#### Scoring Procedure

Subject movements in the freeze monitor apparatus were determined via the Med Associates Video-Tracking and scoring software. Data are expressed as percent freezing in 60-s epochs, each epoch divided into 6 or 12 5-s bins.

### Statistical Analysis

All data were analyzed and graphed using SigmaSTAT and SigmaPLOT software programs, respectively. Open field cumulative counts were compared using 2-way repeated measure ANOVA (genotype vs. time) with pair-wise multiple comparison procedures using Holm-Sidak method. Open filed zone-time and distance traveled data were analyzed by one way ANOVA. Data from fear conditioning experiments were analyzed using two-way repeated measure ANOVA with comparison between wild-type versus knockout and non-transgenic versus transgenic genotypes. Data from hot plate tests (temperature and time) were analyzed using one-way ANOVA. Comparisons producing a p value <0.05 were considered significant.

## Results

### Age-dependent decrease in the expression of αB-crystallin in transgenic mice

**Figure 1:**
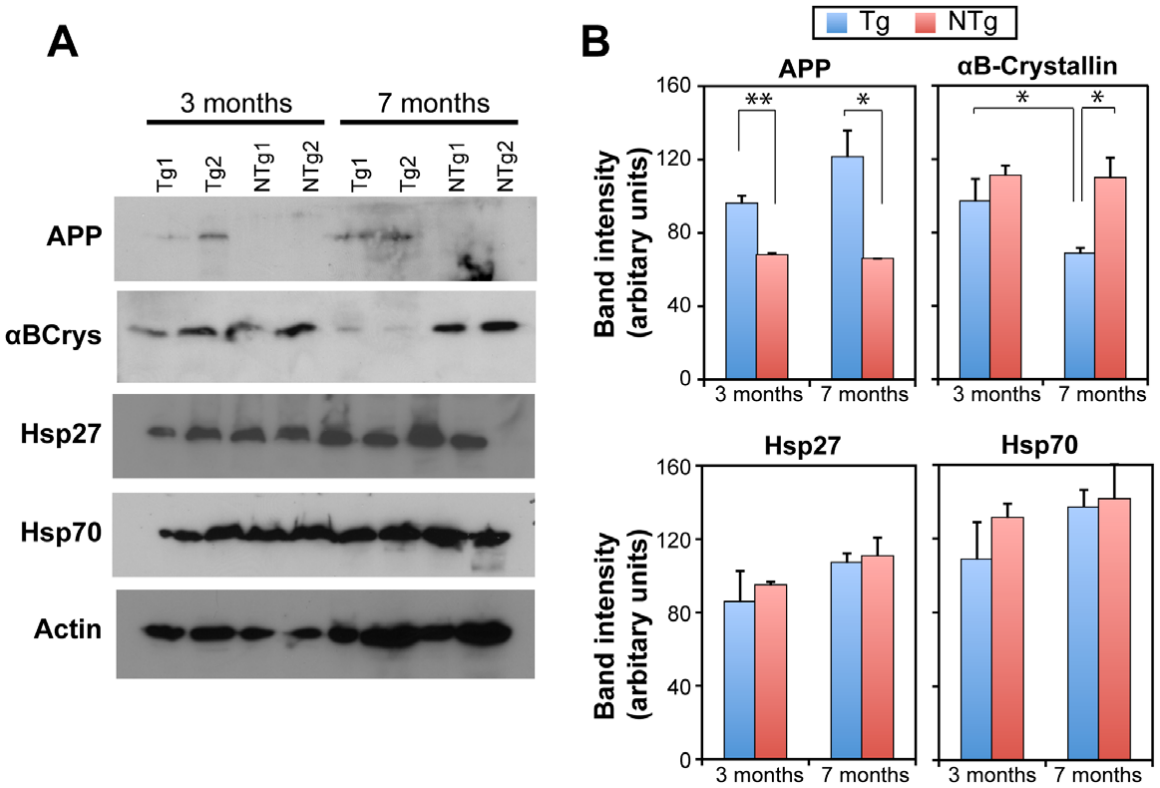
Chaperone levels in AD model mice. (A) Immunoblots showing age-dependence of αB-crystallin expression in mice expressing mutant human APP transgene. Brain lysates were prepared from two individual non-transgenic (NTg1 & NTg2) or transgenic (Tg1 & Tg2) mice each at 3 months or 7 months of age. Samples were immunoblotted for αB-crystallin, Hsp70, Hsp27, Aβ and actin. 40 µg of total protein was analyzed. Similar levels of Hsp70, Hsp27 and actin in all samples show equal protein loading. (B) Densitometric analysis of band intensities in A. Intensities were compared by two-sample Student's t-test for statistical signifcance. APP expression in Tg mice were significantly greater than NTg mice as expected (p = 0.006 at 3 months, p = 0.017 at 7 months). αB-crystallin expression was significantly lower at 7-months compared to 3-months old transgenic mice brain (p = 0.044). αB-crystallin levels in 7-month old transgenic mice was also significantly lower than non-transgenic mice at similar age (p = 0.018). All other comparisons showed no significant differences.

As guardians against protein misfolding and aggregation, chaperones protect the cells from the deleterious effects of aggregation. Stressful conditions such as heat shock cause an acute increase in misfolded proteins resulting in an increase in demand for chaperones, which is normally met by the stress-inducible expression of chaperones. On the other hand, a steady increase in damaged proteins (oxidized and nitrosylated) takes place due to aging, leading to a chronic demand for chaperones and accumulation of damaged proteins. Such a reduction in chaperone availability is thought to play a central role in triggering the protein aggregation cascade of natively unstructured and amyloidogenic polypeptides associated with several neurodegenerative diseases. Because misfolding and aggregation of the amyloid-beta (Aβ) peptide is a major hallmark of AD, we premised that an age-dependent decrease in available chaperones might exacerbate the increased demand for proteostasis mechanisms. We investigated this possibility by examining the expression of Hsp70, Hsp27 and αB-crystallin in the brains of non-transgenic and transgenic mice at young (3 months) and older (7 months) ages by immunoblotting (Figure 1A) and densitometric analysis of the band intensities (Figure 1B). Due to the muscle-specific expression of HspB2 and the unavailability of appropriate antibodies recognizing mouse HspB2, we did not examine its expression in the mouse brain. The expression of the human APP transgene was examined using an antibody specific to human Aβ. As expected, only transgenic mice showed expression of APP and the band intensities were significantly above the intensities in the same molecular weight range in non-trangenic mice (p<0.05, two-sample t-test). The expression of Hsp70 and Hsp27 were unchanged at the ages tested irrespective of the transgene expression or age of mice. A marginal elevation in the expression of Hsp27 and Hsp70 observed in Figure 1B was not significantly different between the two ages or between the two genotypes (p>0.05, two-sample t-test). The equal expression levels of these proteins also serve as control for equal protein loading. Interestingly, the expression of αB-crystallin was significantly decreased in older transgenic mice but not in non-transgenic mice. Densitometric measurement of band intensities showed that αB-crystallin levels of 3-month old transgenic mice were comparable to that in non-transgenic mice. The levels of αB-crystallin in 7 month-old transgenic mice decreased significantly compared to 3-month old transgenic mice (p<0.05, two-sample t-test). This suggests that αB-crystallin may be an early indicator of protein misfolding defects in the brains of transgenic mice.

### Generation of transgenic AD mice lacking αB-crystallin/HspB2

**Figure 2:**
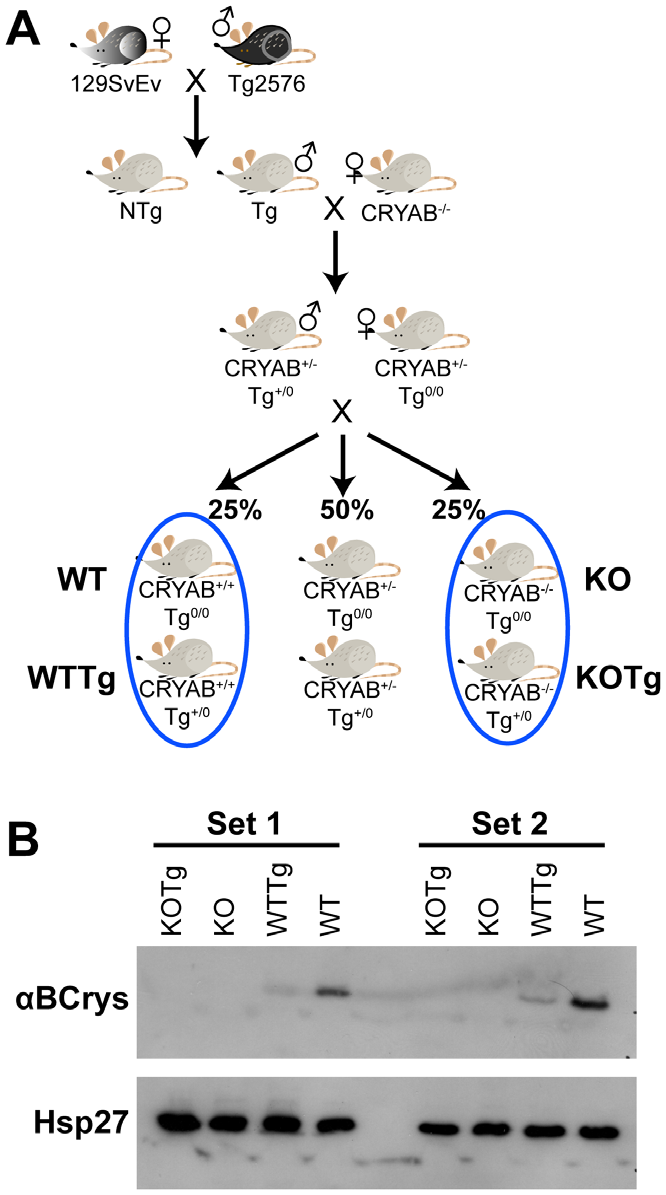
Generation of the required genotypes of mice. (A) Schematic diagram showing the mouse crosses that lead to the required genotypes. (B) Immunoblots showing αB-crystallin and Hsp27 expression in two sets of mice at 7 months of age. WT is *CRYAB^+/+^HspB2^+/+^, Tg^0/0^*; WTTg is *CRYAB^+/+^HspB2^+/+^, Tg^+/0^*; KO is *CRYAB^-/-^HspB2^-/-^, Tg^0/0^* and KOTg is *CRYAB^-/-^HspB2^-/-^, Tg^+/0^*.

Based on our observations of reduced αB-crystallin in older transgenic but not in non-transgenic mice, we hypothesized that if this chaperone is protective, then its loss may show an exaggerated phenotype. To examine if decreased αB-crystallin levels were important for the manifestation of the behavioral phenotypes in the AD model mice we crossed Tg2576 mice (B6/SJL background) with wild-type (*CRYAB^+/+^HSPB2^+/+^*) or αB-crystallin/HspB2 knockout (*CRYAB^-/-^HSPB2^-/-^*) mice (129Sv background). The breeding scheme is shown in Figure 2A. This intercross resulted in litters with equal numbers of non-transgenic (Tg^0/0^) or hemizygous transgenic (Tg^+/0^) mice, which were heterozygous for αB-crystallin/HspB2 (*CRYAB^+/-^HSPB2^+/-^*). The litters were inbred to obtain mice of the following genotypes – (i) *CRYAB^+/+^HSPB2^+/+^, Tg^0/0^*; (ii) *CRYAB^+/+^HSPB2^+/+^, Tg^+/0^*; (iii) *CRYAB^-/-^HSPB2^-/-^, Tg^0/0^* and (iv) *CRYAB^-/-^HSPB2^-/-^, Tg^+/0^*. From this point on, these mice are denoted as WT, WTTg, KO and KOTg, respectively. (Mice heterozygous for CRYAB (namely, *CRYAB^+/-^HSPB2^+/-^, Tg^0/0^*; and *CRYAB^+/-^ HSPB2^+/-^, Tg^+/0^*) that were also generated in this cross were not used further.) By comparing only littermates for all experimental genotypes, we minimized the influence of genetic variation resulting from the parental strains. Further, any residual effects of genetic variation are expected to be evenly distributed between the four genotypes. The chaperone expression in each genotype was confirmed by western-blotting two sets of brain lysates from 7-month old mice for αB-crystallin, and Hsp27 (Figure 2B). The expression of Hsp27 was equal in all mice suggesting that the expression of Hsp27 was not increased to compensate for the loss of αB-crystallin/HspB2. The KO and KOTg samples were devoid of αB-crystallin as expected. WT samples showed higher amounts of αB-crystallin than WTTg samples as observed in Figure 1. Expression of APP was observed only in the transgenic samples (KOTg and WTTg) as expected (not shown). Although the WTTg mice express low levels of αB-crystallin, they are distinct from KOTg mice, which are devoid of αB-crystallin since embryogenesis.

### Behavioral analysis

The decrease in αB-crystallin expression in older transgenic mice brains suggested that the over-produced APP may affect proteostasis not only by increased demand for chaperone function but also reduced expression. We premised that if αB-crystallin protected the mice from Aβ-mediated neuronal dysfunction, then in its absence these deficits should manifest at an earlier age. It was demonstrated that the Tg2576 mice showed behavioral changes as early as 6–7 months of age [34]. Since the mice deficient for αB-crystallin/HspB2 showed debilitating phenotypes about 9-10 months of age [32], we decided to investigate the behavioral phenotypes of WT (n = 9), WTTg (n = 7), KO (n = 15) and KOTg (n = 7) mice when they were 7−8 months (or 28–32 weeks). The following tests were designed to examine both the effects of Aβ on locomotion defects in αB-crystallin/HspB2 deficient mice and the effects of αB-crystallin/HspB2 loss on memory network defects in Tg2576 mice.

#### Open field Test

Open field test is one of the most common tests to monitor general motor activity, exploratory behavior and measures of anxiety [35]. Normal behavior in mice is to seek the protection of the periphery rather than the vulnerability of the center. Mice that are less anxious are expected to spend more time in the center. Mice that show signs of motor deficits are expected to show lower horizontal distances and vertical movements. Mice were individually placed in an open arena equipped with infrared photobeams to monitor mice behavior for 30 minutes without the experimenter being present in the room. The photobeams recorded the following activities – (i) counts of ambulatory (walking), stereotypic (repetitive activity, eg, grooming) and vertical (rearing up) movements; (ii) distance travelled in the center versus periphery and (iii) zone times in center versus periphery were monitored.

**Figure 3:**
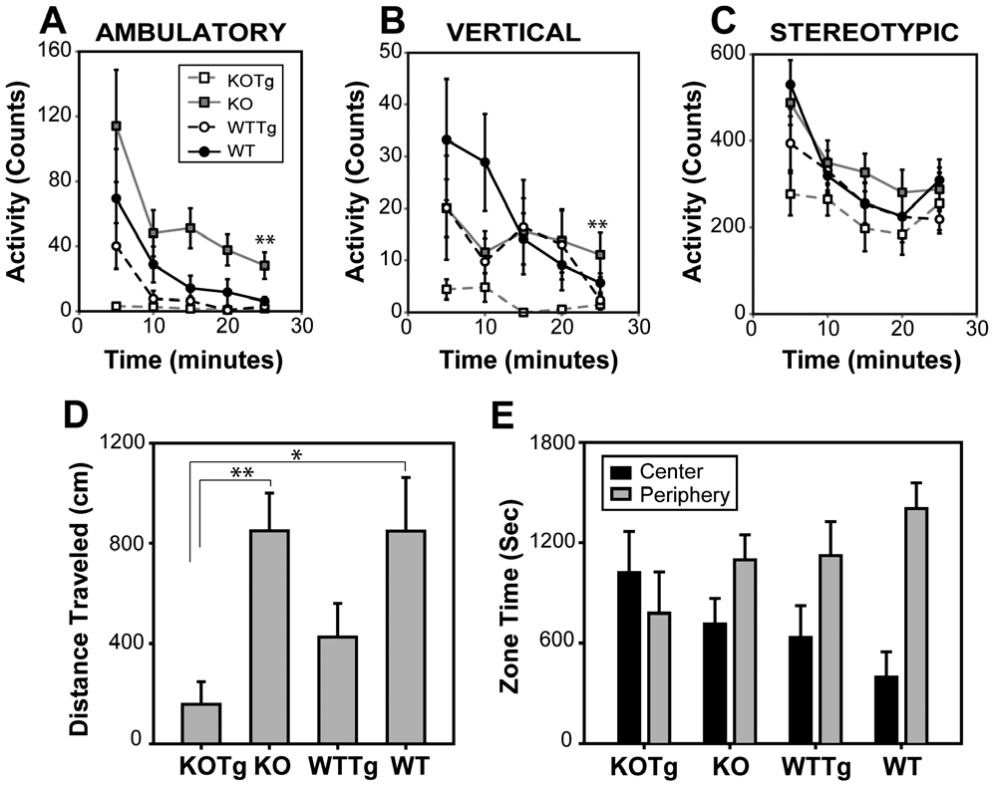
Open field test. Counts for Ambulatory (A), Vertical (B) and Stereotypic (C) activities of mice over a period of 25 minutes in the open field. Symbol representations are - KOTg (open grey square; n = 7), KO (closed grey square; n = 15), WTTg (open black circle; n = 7) and WT (closed black circle; n = 9). Mean values ± SEM are plotted. Data were analyzed by two-way repeated measure ANOVA, which indicated that KOTg group showed significantly less (p = 0.004) than other groups. Differences were not statistically significant between other groups. (D) Total distance (in cm) traveled by the mice over a period of 30 minutes. Mean values ± SEM are plotted. Data were compared by t-tests. KOTg was significantly different from KO (p = 0.007) and WT (p = 0.017). (E) Zone time in center (black bars) and periphery (grey bars) for the mice during the 30 minutes. Data were compared by t-tests. Center zonetimes of both KO and KOTg were significantly different from WT (p = 0.023 for KO and p = 0.0006 for KOTg). Peripheral zonetimes were similar for all groups. (n = 7 for KOTg; n = 15 for KO; n = 7 for WTTg and n = 9 for WT).

First of all, WT and KO groups behaved similarly in terms of vertical counts, stereotypic counts and ambulatory counts indicating that loss of αB-crystallin/HspB2 alone had negligible effect on locomotion at this age group (Figure 3A, B & C). Age associated muscle degeneration reported with the knockout mice was not manifested in terms of locomotion defects at this age group as all 3 parameters counts of KO group of animals was comparable to WT. Similarly, the WTTg group was comparable to WT suggesting that the expression of mutant APP in mice did not significantly affect locomotion by itself. Interestingly, when the transgene expression was combined with αB-crystallin/HspB2 deficiency in the KOTg group, the effect was synergistically enhanced compared to KO group (P<0.004, 2-way repeated measure ANOVA) in ambulatory activity and vertical activity. Differences were not statistically significant in stereotypic activity.

The total distance traveled by WT and KO mice were also very similar underscoring the lack of locomotion deficits in αB-crystallin/HspB2 knockout mice at this age. Distance traveled (Figure 3D) and zone times in the center and peripheral regions (Figure 3E) of the open field were also comparable (differences were not statistically significant). Although the total distance traveled by WTTg mice was significantly lower than the WT group, the zone times and distance traveled in the center and periphery were statistically similar. Similarly, KOTg mice traveled only about a third the distance traveled by KO mice but differences between center and periphery were not significant. Total distance traveled by KOTg mice was also significantly lower than that of WTTg mice highlighting the synergistic effects observed in ambulatory activity counts (Figure 3A). These results suggest that the differences between transgenic (WTTg and KOTg) and non-transgenic (WT and KO) mice were due to locomotion defects rather than defects in anxiety or exploratory behavior. The KOTg mice appeared to be unable to distinguish between center and periphery compared the WT group (Figure 3E) suggesting that they were significantly less anxious than WT mice. Although, direct comparisons between KOTg and WT groups are complicated, our data suggests synthetic effects of αB-crystallin/HspB2 deficiency and APP transgene expression.

#### Hot plate test

**Figure 4:**
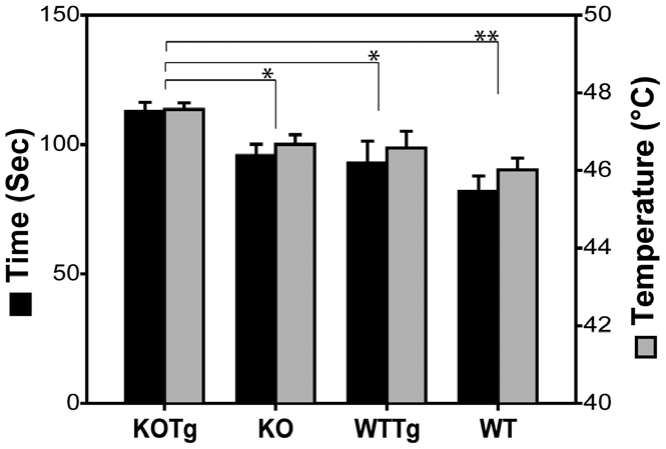
Hot plate test. The duration of time to elicit a thermo-sensitive reflex response in mice is shown on the left axis (black bars). The temperature at which the response was recorded is shown on the right axis (grey bars). Mean values ± SEM are plotted. Data for different groups were compared by t-tests. Both the time and temperature required elicit a response in KOTg were significantly higher than those for KO (p = 0.022 and 0.031, respectively), WT (p = 0.001 and 0.001, respectively) and WTTg (p = 0.05 and 0.05, respectively). All other groups were statistically similar. WT is *CRYAB^+/+^, Tg^0/0^*; WTTg is *CRYAB^+/+^, Tg^+/0^*; KO is *CRYAB^-/-^, Tg^0/0^* and KOTg is *CRYAB^-/-^, Tg^+/0^*. (n = 7 for KOTg; n = 15 for KO; n = 7 for WTTg and n = 9 for WT).

To understand whether the observed locomotion defects were coupled with sensory defects, we examined the mice for pain sensitivity to thermal stimulus (nociception). Each mouse was placed on a hot plate and the temperature of the heating surface was raised by 3°C per minute. The minimum time and temperature required to elicit response (paw licking or jumping) was noted (Figure 4). Somatosensation and its response as motor activity were scored to test for spinal cord related reflexes. The KO, WTTg and WT mice showed comparable responses and the differences were not significant. Maximum latency and higher temperatures were required for the KOTg group to elicit a response and were significantly greater than all other groups of mice with respect to both time and temperature. This result suggests that the combined effect of αB-crystallin/HspB2 loss and transgene expression caused a decreased sensory function not observed with each genotype individually, thus underscoring the synergism.

#### Fear conditioning test

**Figure 5:**
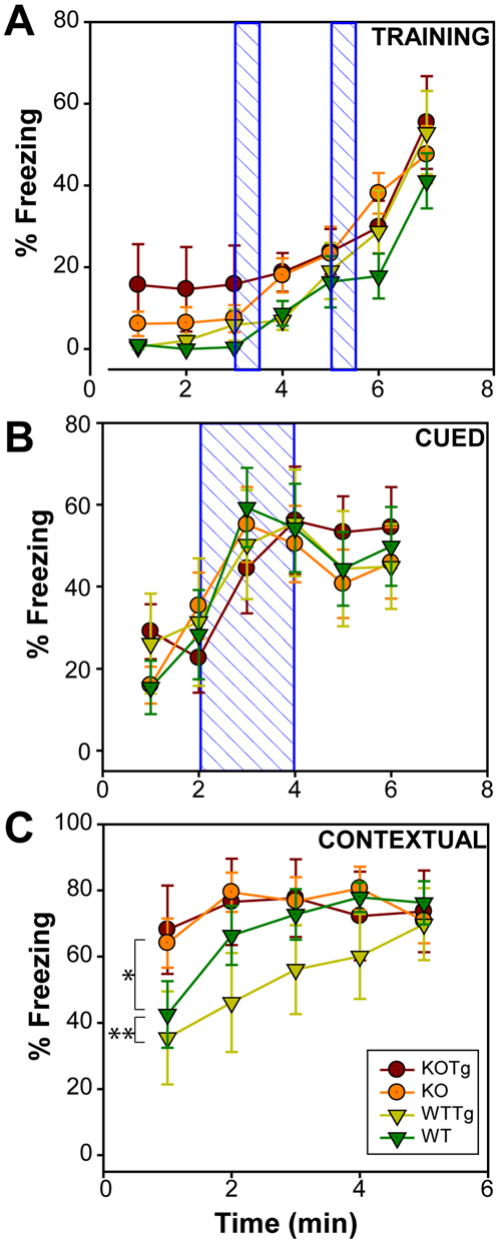
Fear conditioning test. Percent freezing of mice over time during Training (Top), Cued test (middle) and Contextual test (bottom). Mean values ± SEM are plotted. Data were analyzed by two-way repeated measure ANOVA. Differences between groups were not significant for training and cued tests. In contextual tests, WT performed significantly better than WTTg (p = 0.003); difference between WTTg and KOTg was significant (p = 0.014); other groups were not significantly different. Blue hatched boxes represent two separate auditory signals (80dB) of 30 seconds each during training and a single auditory signal for 2 minutes during cued test followed by 2 sec foot shock. Symbol representations are - KOTg (red circle), KO (orange circle), WTTg (yellow triangle) and WT (green triangle). (n = 7 for KOTg; n = 15 for KO; n = 7 for WTTg and n = 9 for WT).

In order to examine whether learning behavior was impaired in transgenic mice lacking αB-crystallin/HspB2, we performed fear-conditioning test. In this test paradigm the associative learning of a neutral cue (eg, sound tone) or a neutral context (eg, environment) with a brief aversive stimulus (eg, mild electric shock) is measured by monitoring the freezing behavior in mice (Figure 5). Fear conditioning test was performed by placing a mouse in a box equipped with a mechanism for monitoring the freezing behavior of the animal by recording photobeam breaks. The mice were first trained to associate the surroundings or a sound pulse (cue) with the mild aversive stimulus. Cue-dependent freezing was tested in a novel environment (i.e., one with different lighting, and olfactory and visual cues) and the freezing behavior associated with the tone was measured. Context-dependent freezing was monitored to evaluate the learned aversion of an animal for the environment associated with the mild aversive stimulus.

No significant differences in freezing behavior were obvious during the training period (Figure 5A) indicating that the locomotion defects observed with WTTg and KOTg mice (Figure 3) did not significantly affect the freezing response to the mild aversive stimulus. During training, mice did not exhibit freezing during the first cue-foot shock pair at 3 minutes, but freezing increased in all mice during the second cue-foot shock pair at 5 minutes. The subsequent increase in freezing at the end of 6-minute period suggested an anticipatory behavior for a third cue-foot shock pair or could also suggest decreased exploratory behavior due to familiarity with the surrounding.

In response to a tone cue (Figure 5B), the mice behaved similarly with no significant differences in the freezing behavior. Mice did not exhibit freezing and explored their novel environment during the first 2 minutes. All mice responded readily to the tone cue presented to them for 2 minutes by freezing. The mice mostly stayed frozen beyond the 4-minute time point. These results indicated good associative learning in all mice.

Interestingly, in response to contextual associative learning there were notable differences (Figure 5C). First, WT and WTTg mice froze similarly at the start of the experiment but differed significantly at later time points (p = 0.003). While the WT mice rapidly froze subsequently and reached maximum freezing at the 2-minute time point, the WTTg mice only showed a gradual increase in freezing till the 5-minute time point. This suggested that context-dependent associative learning may be one of the first manifestations of APP transgene expression. Second, KO and KOTg mice showed a high degree of freezing behavior from the start of the experiment in comparison to their wild-type counterparts. This is an intriguing result – mice lacking αB-crystallin/HspB2 mice were seemingly better at contextual learning than their wild-type counterparts. This result cannot be satisfactorily explained by the locomotion defects in the knockout mice because the same mice are normal in the cued experiment. This leads us to suggest that the mice lacking αB-crystallin/HspB2 were perhaps hyper-sensitive to a particular contextual signal (such as auditory, visual, olfactory or tactile), which may have allowed them to associate this signal as a strong unitary cue with the aversive stimulus. The nature of this cue is unclear.

## Discussion

In this study, we aimed to investigate the effect of chaperone-deficiency in a mouse model for AD. When expression of APP was combined with loss of αB-crystallin/HspB2, we observed that new phenotypes involving locomotion and sensory function deficits were revealed. Neither loss of αB-crystallin/HspB2 nor transgenic expression of APP by themselves produced these phenotypes. The synthetic sick phenotypes underscore a negative synergy between expression of APP, which is thought to increase the production of the aggregation-prone Aβ peptide and reduced chaperone (αB-crystallin and HspB2) function.

When misfolding-prone proteins are overproduced, cellular health may depend on its ability to also overproduce chaperones. In AD brains, where the load of misfolded proteins is high, others have observed that αB-crystallin expression was high in glial cells in areas surrounding plaques and tangles [20], [36]. However, we observed that αB-crystallin levels in brain lysates decreased in an age-dependent manner in transgenic AD model mice compared to non-transgenic littermates. The contrasting observations may be attributed to the differences in methods - while others have monitored relatively small regions by immunohistochemistry in human AD brains [20], [36], we monitored the expression levels by immunoblotting lysates from the entire brains of a mouse model for AD. In this regard, we would like to point out that other studies have observed no significant differences in the expression of αB-crystallin in various regions of AD brains by immunoblotting methods [37], by mass spectrometry based protein identification methods [38] and by immunohistochemical methods [39]. Further, we speculate that the difference between the human and mouse results could be due to differential cellular responses towards the entire gamut of pathologies in AD brains versus the predominantly plaque pathology in the AD model mice used in this study. However, the exact reasons for these observations are presently unclear and require further experimentation.

We were unable to examine the effect of αB-crystallin/HspB2 loss on the deposition of Aβ plaques in the mice because the KOTg and the KO mice were considerably debilitated by the age of 9–10 months as previously reported for αB-crystallin/HspB2-deficient mice [32]. At this age, WTTg mice do not have significant levels of plaque pathology and plaques develop only in mice older than 10-12 months of age [34]. Immunohistochemical examination of mice brains at 7 months revealed no plaques under our experimental conditions (data not shown). However, it is thought that plaques may not be causative and others have shown that decreased dendritic spine density, impaired long-term potentiation (LTP), and behavioral deficits occurred many months before detectable plaques [40].

We observed a modest locomotor deficit in WTTg mice by open field tests (Figure 4D) suggesting that the expression of hAPP affected muscle function. In Tg2576 mice, expression of the APP transgene has been observed in muscle tissue [41] where the function of αB-crystallin and HspB2 is expected to be crucial [32]. The presence of amyloid oligomers in muscle cells has been shown to have potent toxicity [42]. Thus, the high degree of synergistic toxic effects of αB-crystallin/HspB2 loss and transgene expression in the skeletal muscle precluded the examination of plaque pathology at later ages. Because sHsps block the fibrillization and toxicity of Aβ [43], [44] it is suggested that in the absence of αB-crystallin/HspB2 enhanced Aβ toxicity may contribute to the manifestation of the synergistic phenotypes. An alternative explanation for the locomotion defect is that since APP is expressed throughout the brain in this mouse, it might contribute to deficits in the brain motor centers. The locomotion defects observed in the WTTg mice are important because these transgenic mice are routinely used in learning and memory tests that involve locomotion (eg, swimming in water maze). In agreement with our observations, motor deficits in Tg2576 have been observed at 6–7 months in a recent publication [45]. No significant perturbation in the motor reflexes was observed in WTTg mice as the mice could perceive and respond to thermal stimulus in the supraspinal nociception (hot-plate) test. However, reduced chaperone levels in the transgenic mice produced a sensory defect suggesting that this synergism also affected nociception.

In the WTTg mice, we also observed a significant limitation in their ability to associate mild aversive stimulus with the environmental context but not with an auditory cue. This difference in associative learning can be interpreted as follows – Contextual learning may be based on weak associations between the footshock and diverse signals (including visual, olfactory, auditory and sensory stimuli) and thereby differences between WT and WTTg are manifested significantly [46]. It has been thought that animals may associate only a subset of contextual elements, which leads to weak associative learning. The inability of WTTg to associate and integrate the complex contextual signals with the aversive stimulus indicates a reduced hippocampal function. Contextual associative learning deficit in Tg2576 has been previously observed [40], [45]. On the other hand, associative learning may be reliably paired with footshock when a strong unitary cue (such as an auditory tone) is provided and a Pavlovian response is established [47], which may overcome the minor differences in learning between different genotypes. The ability of WTTg mice to normally associate the fear of footshock with an auditory cue indicates that there is no obvious impairment in amygdala under these conditions. An alternative explanation for the superior contextual learning in KO and KOTg mice is a possibility that αB-crystallin and/or HspB2 may be involved in the folding or degradation of proteins that modulate this learning.

Because molecular chaperones prevent excessive misfolding and aggregation, cellular demand for chaperones are greater in aged and stressed cells, which accumulate damaged proteins. Our results demonstrating synergistic effects of reduced chaperones in the context of a mouse model for AD highlights the in vivo importance of sHsps in diseases characterized by protein misfolding and aggregation.
